# A Hybrid Model for Ultrasound Image-Based Breast Cancer Diagnosis Using EfficientNet-V2 and Vision Transformer

**DOI:** 10.3390/diagnostics16081176

**Published:** 2026-04-15

**Authors:** Zainab Qahtan Mohammed, Amel Tuama Alhussainy, Ihsan Salman Jasim, Asraf Mohamed Moubark

**Affiliations:** 1 Department of Computer Science, College of Basic Education, Diyala University, Diyala 32001, Iraq; drihsan@uodiyala.edu.iq; 2 Department of Artificial Intelligence Engineering Techniques, Technical Engineering College of Computer and AI/Kirkuk, Northern Technical University, Kirkuk 36001, Iraq; amel.tuama@ntu.edu.iq; 3 Department of Electrical, Electronic and Systems Engineering, Faculty of Engineering and Built Environment, University of Kebangsaan Malaysia, Bangi Selangor 43600, Malaysia

**Keywords:** breast cancer, ultrasound, Efficient Net, vision transformer, hybrid model, classification

## Abstract

**Background/Objectives**: Breast cancer continues to be one of the most serious and common afflictions affecting women around the globe. Despite ultrasound imaging being an effective method for the detection of abnormalities in dense breast tissues, there are a number of drawbacks when utilizing this method, including the subjective nature of the imaging and the variant nature of the imaging due to the cognitive biases of the interpreting expert and the experience of the interpreting expert. The above factors are the cause of the increased need in the implementation of AI-driven models for diagnostic analysis. In this research, we provide a hybrid deep learning-based framework for cancer classification of the breast cancer ultrasound image dataset (‘BUSI dataset’). **Methods:** The contributing models of the proposed architecture involve the combination of a light ViT encoder and an EfficientNetV2-RW-S feature extractor. The combination mentioned leverage the positive sensitivities of the convolutional neural networks (CNNs) and the global reasoning neural networks (i.e., transformers) in the explanation of the architecture. The reason being, EfficientNetV2 diminishes the capture of the fine-grained morphological components of the lesions, edges, and echogenic variances of the tissue, whereas the transformer model diminishes the long-range dependencies of the lesions and other surrounding tissues. **Results:** The experimental results from the proposed hybrid model of the architecture demonstrates an enhanced classification accuracy of 97.95%, in contrast to the self-standing models of the architecture, the hybrid model supersedes the isolated ViT model (i.e., 89%) and the isolated CNN model (i.e., 80%) frameworks. Furthermore, the proposed model hybrid architecture also diminishes the overall self-attention computational complexity of the proposed model by substantially diminishing the number of tokens reaching an overall count of 10 (from the vast 197 tokens). This further leads to a substantial decrease in the memory and cost expended during the attention processes. **Conclusions:** Overall, this study proposes a method for the improved diagnostic and computational analysis, suggesting the proposed architecture to be a potential framework for use in the contemporary clinical environments.

## 1. Introduction

Breast cancer is a persistent problem for the medical field and millions of women around the world. According to the American Cancer Society reports, 1 in 8 women in the U.S. will be diagnosed with breast cancer in their lifetime [1]. This underscores the pressing necessity for efficient cancer detection and treatment methodologies. The number of cases emphasizes the urgency to develop better methods for detecting and treating the disease. Diagnostic ultrasound imaging represents a primary modality for breast cancer screenings and diagnostic evaluation. It is affordable, safe, and can capture images in real time. However, diagnostic ultrasound still relies on subjective and inconsistent manual interpretation from the diagnostician. Because of this, the same ultrasound can produce different diagnoses and therefore delay treatment. This reiterates the need for the development of tools that can objectively and consistently analyze ultrasound studies so that clinicians can focus their efforts on tools that enhance the reliability of such systems.

Several aspects of analysis in medical imagery are enhanced and refined by deep learning techniques, with a specific emphasis on convolution neural networks CNNs. These methods involve the stacking of convolutional and down-sampling layers, often incorporating residual connections. CNNs have the ability to capture and hierarchically describe different attributes of medical images. However, the CNN algorithms are constrained because they work on the basis of local operations, implying that they lack the ability to capture long-range dependencies. Furthermore, as layers grow deeper, increased abstraction lessens the likelihood of retaining significant information; that is, they lose important spatial and contextual information. Consequently, their vision-task applicability is constrained by the local receptive fields of the algorithms. In recent years, numerous attempts have been made to integrate deep learning across multiple fields owing to the rapid capture of long-range information made possible by the transformer model. In contrast to CNNs, which operate on a local receptive field basis, vision Transformers (ViTs) operate on a global receptive field basis by leveraging self-attention mechanisms [2]. In spite of this, the effectiveness of these approaches is constrained by the abundance of data required to form meaningful representations of images. The ViT architectures lack the intrinsic inductive biases such as spatial invariance and locality, which have made CNNs proficient at spatial feature extraction in images.

EfficientNet is a family of CNNs that have been designed to scale across different dimensions of neural networks. Due to EfficientNet’s compound scaling methodology, networks can be allocated resources in a manner across the different dimensions of the networks which results in improved performance relative to other CNNs with fewer parameters. Given the size of the BUSI dataset, utilizing a frozen EfficientNetV2 that helps to reduce the computational overhead as it reduces the number of parameters that need to be trained. EfficientNet is a good choice for feature extraction due to its ability to extract rich and discriminative features from ultrasound images [3,4]. The architecture captures multi-scale morphological features such as lesion texture, shape, and boundary definition, which makes it well-suited for ultrasound analysis. The vision transformer ViT employs an attention mechanism to determine the importance of far-apart regions of an image in order to understand the structural and contextual relations [5,6].

The hybrid model has been developed combining EfficientNetV2-RW-S and ViT, taking advantage of the strengths of both CNNs and ViTs and proposing the first architecture of this kind tailored to improve classification performance on constrained and variable datasets, particularly unbalanced and low-contrast datasets such as BUSI. In contrast to most ViT models that rely on standard patch tokenization, the proposed model opts for compact tokenization of pooled feature maps whereby the spatial structure is retained and the number of tokens is reduced for the sake of efficiency in memory and computation. This integrated approach allows the model to take advantage of both the fine-grained representation power of EfficientNetV2 and the global reasoning of the Transformer, thereby improving metrics such as precision, recall, and discrimination. In the case of the hybrid framework under the specified imaging conditions, it is balanced in sensitivity to malignant lesions and noise resilience, and in computation efficiency, it is balanced in sensitivity to malignant lesions and noise resilience, and in computation efficiency, it is balanced in sensitivity to malignant lesions and noise resilience, and in computation efficiency. Overall, this framework is balanced in sensitivity to malignant lesions and noise resilience, and in computation efficiency.

**Figure 1:**
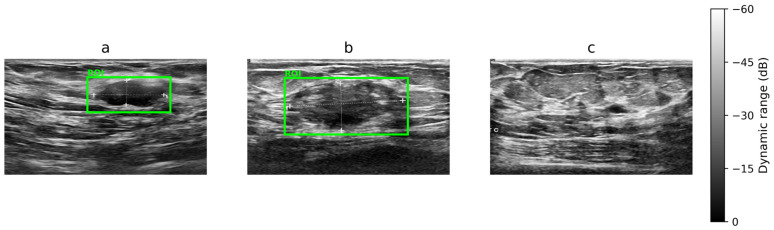
Representative BUSI ultrasound images. Lesion regions of interest (ROIs) are indicated in green in (**a**) benign and (**b**) malignant cases, whereas (**c**) represents the normal condition with no visible lesion.

The proposed framework was validated using the Breast Ultrasound Images (BUSI) dataset, which contains three classes—benign, malignant, and normal—collected from real clinical settings using high-resolution ultrasound systems. Figure 1 presents representative samples from each class; notably, benign and malignant images can appear visually similar in terms of texture and boundary patterns, which makes discrimination challenging and motivates automated analysis. Within this context, the present study contributes a practical hybrid deep learning model that couples local morphological feature extraction with global contextual modeling. As a result, the architecture mitigates known limitations of standalone CNNs and standalone transformers, while maintaining an efficient computational footprint and providing interpretable outputs that are suitable for near real-time clinical decision support.

The contributions of this study are as follows: The proposed approach employs feature-level transformer tokenization in substitute of raw image patches, facilitating more compact and informative representations. It additionally integrates a noise-aware architecture that utilizes speckle-derived texture information in ultrasound images. A lightweight transformer utilizes a limited number of pooled tokens, shallow depth, and compact embeddings, thereby decreasing computing expenses while preserving superior classification efficacy.

## 2. Related Work

In the past few years, deep learning has proven to be successful, especially with CNN-based frameworks like EfficientNet, ResNet, and VGG, in medical image analysis. When it comes to breast cancer detection, these standard deep learning models have produced some fairly positive results. That said, the performance would still be considered hindered due to the typical obstacles of small, unbalanced datasets, which ultimately hurt the generalization and robustness.

Among the various architectures, EfficientNet has achieved notable recognition as it has successfully diagnosed breast cancer in ultrasound images. Using compound scaling, EfficientNet manages to find the sweet spot of accuracy to a given level of computing resources, improving classification while keeping the training and inference costs low. Sahu et al. built a breast cancer detection framework using deep learning and Efficient Net, which utilized uniform and adaptive scaling to show superior classification performance and optimal computational efficiency [7]. Ferreira et al. also reported Efficient Net as having the best performance out of numerous competing SOTA networks on breast lesion classification tasks [8].

Among all variants, Efficient Net V1 and V2 exhibit the most potential for feature extraction from ultrasound images. Banerjee et al. examined EfficientNet V1 (B0–B7) and EfficientNet V2 (B0–B3) for the breast cancer detection and proposed the CEIMVEN Framework [9]. Also, the feature extraction capability and cancerous area identification accuracy are improved by employing transfer learning with the pre-trained Efficient Net model, as stated by Oyebanji et al. [10]. Efficient Net’s compound scaling strategy is the most effective, as it requires fewer resources than most CNNs; thus, training and deployment are faster to achieve.

Despite the strong performance of CNNs, they primarily focus on local feature extraction and often struggle to capture global contextual information. Vision transformers (ViTs) have emerged as a powerful alternative, capable of modeling long-range dependencies through self-attention mechanisms. Dosovitskiy et al. introduced the vision transformer architecture, demonstrating its ability to extract global representations and achieve competitive performance in image classification tasks [11]. Gheflati et al. further demonstrated the effectiveness of ViT models in breast ultrasound nodule classification [12].

Subsequent studies have proposed modifications to the original ViT architecture to improve breast ultrasound image classification. Shareef et al. introduced a hybrid multitask deep neural network, Hybrid-MT-ESTAN, which combines CNNs and Swin Transformer for simultaneous classification and segmentation of breast ultrasound images [13]. Alruily et al. reported that progressive fine-tuning of ViT models achieved an accuracy of 94.49% and an AUC score of 0.921, demonstrating their effectiveness in breast ultrasound image classification [14]. Additionally, the Swin Transformer-based Fork Network (SW-ForkNet) achieved 93.12% accuracy on the BUSI dataset [15]. However, ViT-based models typically require substantial computational resources and large-scale datasets, limiting their suitability for real-time medical deployment.

In the battle of standalone models, CNN and ViT, most recent research aims to construct hybrid models and frameworks of the two to further improve the accuracy of diagnoses.

Anari et al. proposed Efficient-UNet, a model that also has a paired, pre-trained vision transformer and has a breast tumor segmentation model [16]. Other models, hybrid ones too, of the style CNNs with TokenMixer and DFViT, have performed better when it comes to the localization and classification of tumors [17,18].

In addition, the integration of transformers has shown a reduction in the negative impact of noise and variability in the ultrasound images, which in turn leads to an improved and more robust classification. Wu et al. proposed C-TUnet, a hybrid CNN—Transformer network, to classify breast ultrasound images, and it has shown improvements when compared to the previous methods on the public datasets [19]. Tagnamas et al. used a multitask approach that incorporates CNNs and transformers for the segmentation and classification of breast tumors, and it showed improved accuracy and clinical utility [20]. For Efficient Net–ViT hybrid models, there still remain complexities of an architecture and a deficit of a large training dataset. In addition to this, the successful clinical deployment of models will require a lot of rigorous testing in order to validate their clinical applicability to ensure consistency in a wide variety of clinical environments.

Building upon these findings, the present study proposes a two-stage hybrid architecture that integrates EfficientNetV2 with a vision transformer. EfficientNetV2 is employed to extract spatially rich and compact feature representations, which are subsequently reshaped into tokens and processed by the ViT for global relational modeling. This design ensures improved alignment between feature extraction and classification, resulting in enhanced performance and robustness for breast cancer classification using ultrasound images. The main objective of this work is to develop a reliable and efficient breast cancer diagnosis framework that balances precision and computational efficiency, making it suitable for real-time clinical applications on the BUSI dataset.

There has been a lot of fast development in hybrid deep learning techniques. However, a prior research study has yet to propose constructing hybrid frameworks with EfficientNetV2-RW-S and vision transformer for breast ultrasound imaging classification using the BUSI dataset. Thus, the designed framework is one of a kind and contributes to the fast and automated breast ultrasound imaging cancer diagnosis.

## 3. Materials and Methods

### 3.1. Dataset Characteristics

In this study, we assess the BUSI (Breast Ultrasound Images) dataset, which has been methodically developed for the purpose of the classification of breast tumors. It contains scans from Baheya Hospital, Egypt, totaling 780 images with patients aged between 25 and 75. These images are divided into three categories: benign, malignant, and normal. In detail, the dataset has 437 benign tumors, 210 malignant tumors, and 133 normal breast tissues.

In the area of medical imaging, the BUSI dataset, which is freely accessible, is a standard dataset for breast ultrasound image analysis [21]. Detailed ultrasound images are the primary data source for the BUSI dataset. Although ultrasound pictures are a traditional method for screening breast cancers, they can be small and homogeneous, which means the model can overfit. To combat this, data augmentation and transfer learning must be applied. Images in the BUSI dataset are in grayscale, designed to illustrate the texture and density of the breast tissues, and they are in PNG format with a dimension of 500 × 500 pixels. The dataset has extensive potential to aid research in the areas of detecting, segmenting, and classifying tumors and has been valuable in benchmarking deep learning methods.

### 3.2. Data Augmentation

The BUSI dataset plays an important role in helping train deep learning models for breast cancer detection. In this case, data augmentation is an important technique to improve the dataset’s diversity and richness to enhance deep learning models’ performance, especially in cases with small datasets like BUSI. Data augmentation is employed to artificially expand the training dataset by creating modified versions of existing images. To make the training data more varied, the BUSI dataset has been modified in various ways, such as by rotating, flipping, and rescaling [22].

To improve generalization, mitigate overfitting, and increase data diversity, a class-specific data augmentation strategy was employed, with stronger augmentations applied to malignant cases to address class imbalance in the BUSI dataset. Distinct augmentation pipelines were designed for each class. For the benign class, horizontal flipping and minor rotations were used to introduce geometric variability. For the malignant class, more diverse augmentations were applied, including horizontal flipping, rotation, and color jittering (variations in brightness, contrast, saturation, and hue), to enhance diversity and improve the model’s capability to capture complex lesion features. The normal class was augmented using moderate geometric and photometric transformations.

The above augmentation strategies were applied in an offline manner during data preparation, resulting in an expanded dataset of 878 benign, 1054 malignant, and 269 normal images. From this dataset, 702 benign, 844 malignant, and 215 normal images were used for training and validation. A fixed test set was constructed using only original images (20% per class), comprising 176 benign, 215 malignant, and 54 normal samples.

We acknowledge that applying offline augmentation prior to data partitioning may introduce a potential risk of data leakage if augmented samples derived from the same original image are distributed across different subsets. Therefore, results obtained under this setting should be interpreted with caution.

In addition, online data augmentation was applied during training using random horizontal flipping and brightness–contrast adjustments, while no augmentation was applied to validation or test data, ensuring evaluation on original, unaltered images. This targeted augmentation strategy enhances the diversity of the malignant class while preserving clinically relevant image features, thereby supporting the model’s ability to detect challenging cases.

### 3.3. Transfer Learning

Transfer learning has developed as a powerful deep learning technique, especially for medical image interpretation. This method enables pre-trained models on large datasets to be fine-tuned for specific tasks, which is very useful when working with limited medical imaging data. The automated feature extraction abilities of pre-trained models are essential for differentiating between benign, malignant, and normal breast tumors [23]. In our study, we initialize the EfficientNetV2-RW-S backbone with the ImageNet pre-trained weights and fine-tune it, while the transformer classification head is randomly initialized and trained from scratch.

## 4. Proposed Model

The proposed hybrid deep learning framework combines the strengths of a convolutional neural network backbone and a transformer encoder for automated classification of breast cancer in ultrasound images.

**Figure 2:**
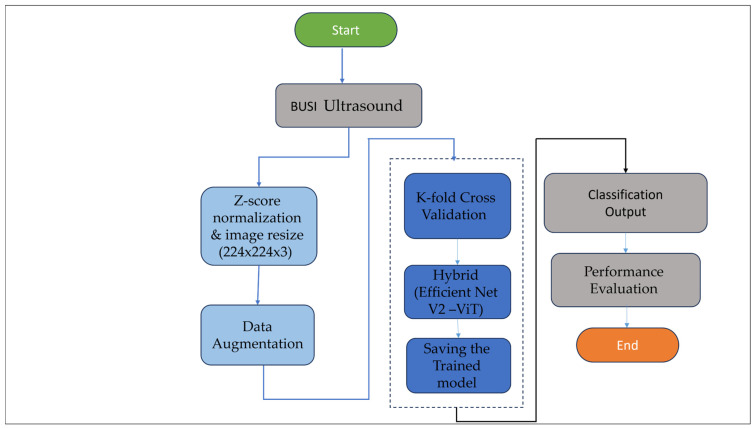
Flowchart of the proposed model. The diagram illustrates the sequential steps involved in the model, acquiring images, normalizing them, partitioning the data, training the model, and finally evaluating it are all shown in the diagram.

Figure 2 shows the overall pipeline of the proposed model, which consists of image acquisition, preprocessing, data partitioning, model training, and evaluation.

The model consists of two main stages: feature extraction using EfficientNetV2-RW-S, followed by a custom transformer encoder based on vision transformer (ViT) principles. This design enables the model to capture fine-grained local features as well as global contextual relationships within the image, thereby improving the differentiation between benign, malignant, and normal tissue patterns.

**EfficientNetV2**

EfficientNet is a family of CNNs designed to scale efficiently across different dimensions of neural networks. The architecture is based on compound scaling, which balances the depth, width, and resolution of the network. This approach allows Efficient Net to achieve state-of-the-art performance while preserving computational efficiency [24].

**Vision Transformer (ViT)**

The ViTs use the transformer architecture. Transformer architecture has been very successful with natural language processing. The self-attention mechanism with vision tasks helps the model understand long-range dependencies and global contextual information. The model outperformed CNN for breast cancer classification, which shows potential as an alternative [25,26].

The suggested hybrid framework uses a convolutional neural network (CNN) with a transformer encoder to facilitate automated classification of breast tumors in ultrasound images. CNNs excel in capturing detailed local attributes (e.g., edges, textures, lesion boundaries) that are critical for defining the morphology of tumors. On the other hand, transformer encoders are good at capturing long-range dependencies and global context across the whole image. The hybrid model combines these two types of strengths, and this enhances the model’s ability to learn more thorough and detailed feature representations, improving the model’s ability to distinguish between benign and malignant tumors in the field of medical image analysis [6,27].

The hybrid framework is designed to enhance classification performance while maintaining computational efficiency and consists of two primary stages:

**Stage 1: Local feature extraction using EfficientNetV2-RW-S**

To meet the input specifications for EfficientNetV2RW-S, all images were adjusted to 224 × 224 × 3 pixels. Fused-MBConv blocks, which are faster and require less memory than regular MBConv blocks, are utilized in the architecture. EfficientNetV2 identifies robust and compact features, notably local features that describe textures, edges, and boundaries of lesions. The compound scaling principle to balanced depth, width, and resolution configuration of the network is incorporated to achieve optimal trade-off between performance and parameter reduction.

**Stage 2: Global feature modeling and classification using Transformer Encoder**

For clinical application purposes, increased emphasis was put on balancing classification and computational efficiency. The proposed model uses EfficientNetV2-RW-S as a feature extractor based on memory saving Fused-MBConv layers and compound scaling.

**Figure 3:**
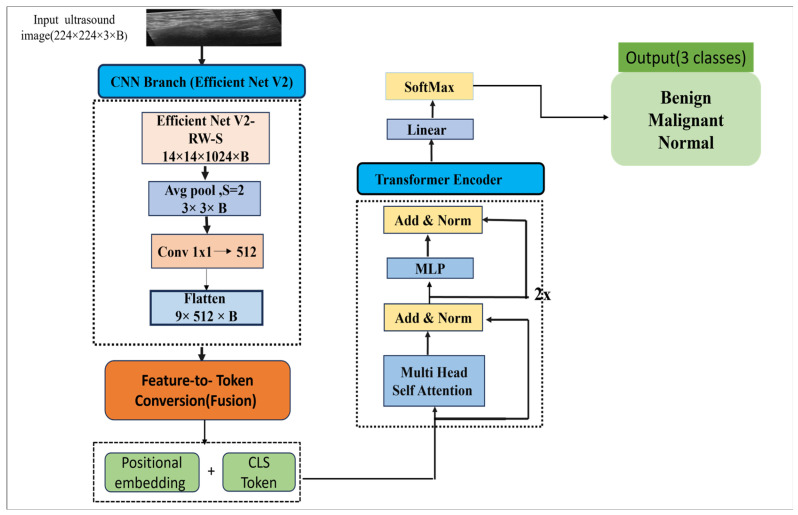
Illustrates overall architecture of the proposed model.

Instead of using raw images for input into the vision transformer (ViT), intermediate features are pools (pooling factor = 2) and then projected into a 512 dimensional embedding using a 1 × 1 convolution. This feature map is then cut into tokens of HxW sequences and a learnable classification (CLS) token is prepended to the sequences. This architecture prevents loss of important spatial information while greatly reducing computational costs. To maintain spatial information, embeddings are added to the input, and a lightweight transformer encoder (2 layers, 8 heads) is added to capture global context of the design. The CLS token is the only token to go through layer normalization and a lightweight multi-layer perceptron (MLP) to obtain the final class logits. The overall architecture of the proposed model is illustrated in Figure 3.

In this context, EfficientNetV2 offers an optimized and powerful backbone for breast ultrasound imaging, aiding in the capture of distinct spatial and morphological characteristics. By compressing the token sequence to 10 elements, a basic ViT encoder reduces the self-attention mechanism’s computational complexity from O(197^2^)to O(10^2^), which enhances the speed of convergence and reduces GPU memory usage.

The hybrid architecture has shown better generalization, illustrating enhanced classification performance and maintaining optimized computational cost in contrast to the EfficientNetV2-only baseline. Model training parameters and system specifications are summarized in Table 1.

**Features Representation Analysis**

The proposed hybrid approach retains local morphological specifics and global contextual details. For feature extraction, EfficientNetV2 develops a hierarchy of visual representations such that the shallow layers learn fine-grained details, while the deep layers learn high-level structures and compositions of breast tissue. The feature maps obtained from these layers are aggregated, tokenized, and sent to a vision transformer (ViT) encoder. The transformer uses multi-head self-attention to simultaneously focus on particular regions of the image and model complex spatial relationships around the lesion. This fused representation allows the model to capture lesion-specific and peri-lesional attributes to better differentiate lesion types.

Clinically, malignant lesions are associated with posterior acoustic shadowing, spiculated or irregular margins, and heterogeneous echogenicity. Benign lesions, on the other hand, are characterized by smooth, well-defined margins and homogeneous echogenicity, while normal tissue has a more or less uniform appearance. The hybrid framework combines these attributes and explains clinical reasoning to distinguish better between malignant, benign, and normal lesions.

## 5. Results

### 5.1. Performance Evaluation

To evaluate the performance of the hybrid model, a comprehensive set of metrics was used, which provides insights into different aspects of classification performance. These metrics include accuracy, precision, recall, F1-score, and macro-F1.

**Accuracy**

Accuracy measures the proportion of true positive predictions among the total number of predictions:

(1) $$Accuracy=\frac{TP+TN}{TP+TN+FP+FN}\;$$

where TP: True Positive, TN: True Negative, FP: False Positive, and FN: False Negative.

**Precision**

Precision (also known as positive predictive value) measures the proportion of true positive predictions among all positive predictions:

(2) $$\mathrm{Precision}\;=\;\frac{TP}{TP+FP}$$

**Recall**

Recall (also known as sensitivity or true positive rate) measures the proportion of true positive predictions among all actual positive instances:

(3) $$Recall=\frac{TP}{TP+FN}$$

**F1-Score**

The F1-score is the harmonic mean of precision and recall, providing a balance between these two metrics:

(4) $$F1\text{-}\mathrm{score}=\frac{2\times \left(Precision\times Recall\right)}{Precision+Recall}$$

**Macro and Weighted Averages**

For multi-class classification, these metrics are calculated for each class separately (class-wise metrics) and then aggregated:

(5) $$Macroa-average=\frac{1}{N}\sum_{i=1}^{N}{Metric}_{i}$$

where ${Metric}_{i}$ denote the evaluation metric for class i, and N is the total number of classes.

(6) $$Weighted-average=\frac{\sum_{i=1}^{N}w_{i\;M_{i}}}{\sum_{i}^{N}w_{i}}$$

Here, *N* denotes the number of classes, M signifies the metric value (precision, recall, or F1-score) for each class, and W represents the number of instances in that class. Macro-averaged metrics assign equal importance to all classes, which is particularly useful for unbalanced datasets, while weighted averages account for class frequencies.

**Matthews Correlation Coefficient (MCC)**

The Matthews Correlation Coefficient (MCC) is a robust evaluation metric that considers all elements of the confusion matrix. It is especially effective for imbalanced datasets, as it offers a balanced measure of classification performance even when class distributions are uneven.

(7) $$MCC=\frac{TP.TN-FP.FN}{\sqrt{\left(TP+FP)(TP+FN)(TN+FP)(TN+FN\right)}}$$

**Figure 4:**
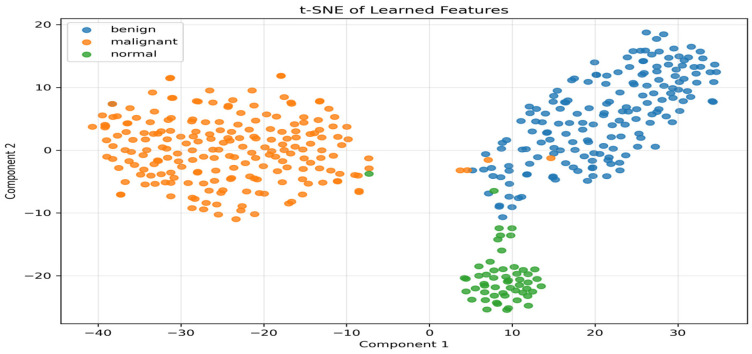
t-SNE visualization of feature embeddings learned by the proposed EfficientNetV2–ViT hybrid model.

The experimental results indicate that the proposed hybrid EfficientNetV2–ViT architecture attains state-of-the-art classification accuracy (97.95%) on BUSI while substantially decreasing Transformer token complexity to 10 tokens, thus reducing computational costs and maintaining diagnostic reliability. The class-specific performance of the best-performing model on the test set, offering detailed diagnostic insights across different tissue types (Table 2). In addition, to ensure robustness, Table 3 presents the mean ± standard deviation derived from the 5-fold cross-validation. Table 4 and Table 5 present the confusion matrix and the computation complexity of the proposed model, respectively. To confirm the hybrid architecture’s effectiveness, t-distributed stochastic neighbor embedding (t-SNE) was utilized on the feature representations to see how discriminately the model worked. The data displayed in Figure 4 illustrates the learned features and the distinct clusters formed for benign, malignant, and normal classes.

This study employs evaluation metrics such as Accuracy, Precision, Recall, and F1-score. All reported Precision, Recall, and F1-score values correspond to weighted averages to account for class imbalance.

### 5.2. Interpretability of the Model Using Grad-CAM

Gradient-Class Activation Mapping (Grad-CAM) has been extensively utilized to clarify classification networks in diverse applications, including breast ultrasound image analysis. This technique makes it easier to understand how well a model works by identifying the region of interest (ROI) based on the gradient score of each class [28].

**Figure 5:**
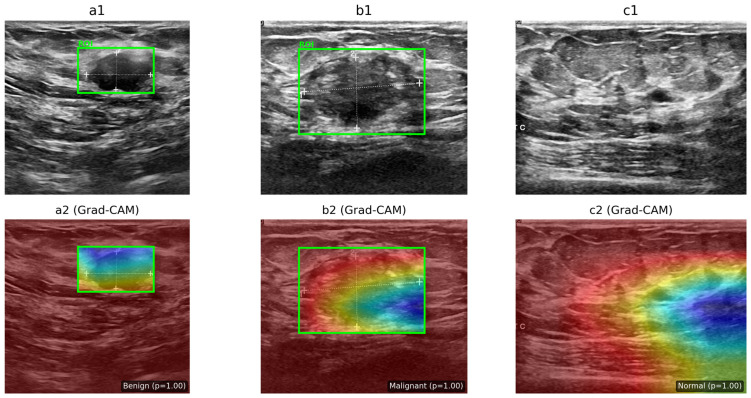
Interpretability analysis using Grad-CAM on BUSI ultrasound images. The upper row shows BUSI ultrasound samples for (**a1**,**a2**) benign, (**b1**,**b2**) malignant, and (**c1**,**c2**) normal cases with radiologist-defined regions of interest (ROI). The bottom row presents the corresponding Grad-CAM heatmaps generated by the EfficientNetV2 feature extractor. Benign and malignant cases exhibit localized activation around lesion boundaries, while the normal case shows diffused activation patterns, indicating the absence of abnormalities.

**Figure 6:**
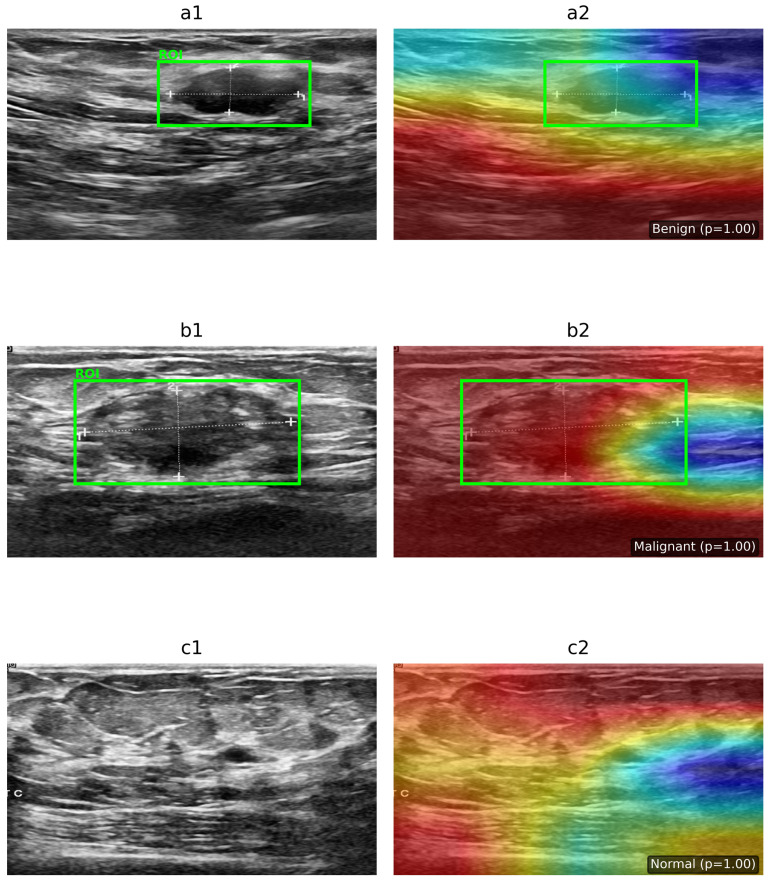
Interpretability analysis using Vision Transformer (ViT) attention maps on BUSI ultrasound images. The left column shows the original ultrasound images with radiologist-defined regions of interest (ROI): (**a1**) benign, (**b1**) malignant, and (**c1**) normal cases. The right column shows the corresponding ViT attention maps: (**a2**) benign, (**b2**) malignant, and (**c2**) normal. The model exhibits localized attention in lesion regions for benign and malignant cases, while the normal case shows more diffuse attention patterns.

To provide interpretability for the proposed hybrid deep learning framework, the Grad-CAM technique was applied to improve model performance by visualizing the regions that contribute most to the final classification decision. Grad-CAM was implemented by attaching forward and backward hooks to the last convolutional layer of the EfficientNetV2 backbone. During forward propagation, feature maps were captured, and in the backward pass, gradients corresponding to the predicted class were extracted. These gradients were globally average-pooled to obtain channel-wise weights, which were then multiplied with the feature maps to emphasize class-discriminative regions. The resulting activation map was rectified using the ReLU function, normalized to the range [0, 1], and up-sampled to match the input resolution (224 × 224 pixels). Finally, a jet colormap was applied, and the heatmap was overlaid on the original BUSI ultrasound image to highlight the regions most relevant to the model’s decision-making. This method made it possible to see how the EfficientNetV2-Transformer worked internally, which made it more transparent and suitable for real-time clinical use, as shown in Figure 5. To address the intrinsic constraint of convolutional networks in capturing long-range contextual dependencies, the proposed hybrid design incorporates a vision transformer as a Global Attention Mechanism (GAM). EfficientNetV2 emphasizes the extraction of localized textural and morphological characteristics, whereas the Transformer identifies global spatial correlations between lesions and adjacent breast tissue. Figure 5 illustrates the attention rollout maps, offering a clear view of global attention dynamics by mapping the information flow from the categorization token through all transformer layers. The attention distributions examined show consistent global alignment with clinically significant areas in both benign and malignant cases, while displaying diffuse patterns in normal images. This affirms that the GAM component helps the model’s ability to think beyond local lesion borders, hence augmenting interpretability and diagnostic reliability as shown in Figure 6.

## 6. Comparative Analysis

The efficacy of the proposed EfficientNetV2–vision transformer (ViT) hybrid model was evaluated against other leading methodologies using the BUSI dataset. The hybrid EfficientNetV2–ViT model was tested on the BUSI three-class problem using a carefully reserved test set composed entirely of original images. The model achieved a malignant-class precision of 99% and an overall accuracy of 98%, with macro-averaged precision, recall, and F1 scores of 98%, 97%, and 97%, respectively.

### 6.1. Benchmarking Against the Literature

To contextualize these findings, we compared our results with four representative studies on BUSI. Our method surpasses the DNBCD framework on BUSI, which achieved an accuracy of 89.9%, while maintaining balanced macro metrics compared to explainable dense-CNN alternatives [29]. Munteanu et al. reported an overall accuracy of 86% on BUSI utilizing a GAN-augmented pipeline [30]. A hybrid CNN-Transformer model (Hybrid-MT-ESTAN) attained 82.7% accuracy [13]. A recent transfer learning benchmark on BUSI, employing classical classifiers with ImageNet backbones, reported accuracy rates of 85.6% for MobileNetV2, 89.7% for ResNet50, and 88.06% for VGG16 [31].

**Figure 7:**
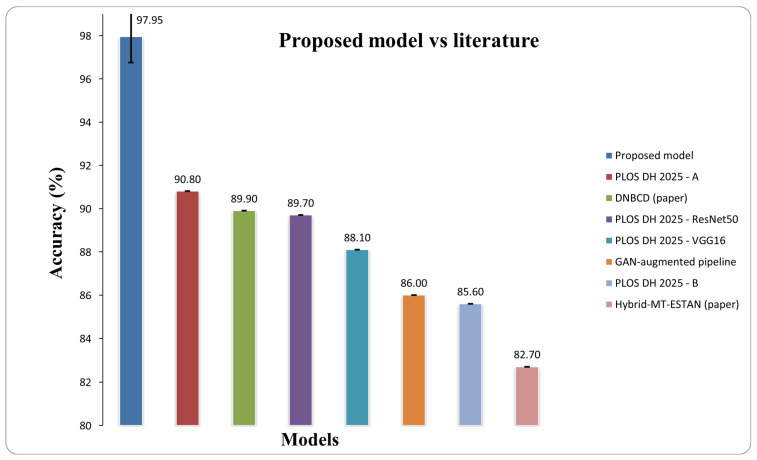
Comparative results of our proposed model vs. Others.

In our controlled evaluation, using a fixed class-balanced test list and matched preprocessing, the proposed hybrid EfficientNetV2–ViT achieved an accuracy of 97.95%, outperforming the best recorded BUSI results by 7.2 percentage points, demonstrating that the fusion design consistently improved performance rather than being influenced by data-splitting artifacts. The comparative performance is illustrated in Figure 7.

### 6.2. Controlled Benchmarking Against CNN Baselines

**Figure 8:**
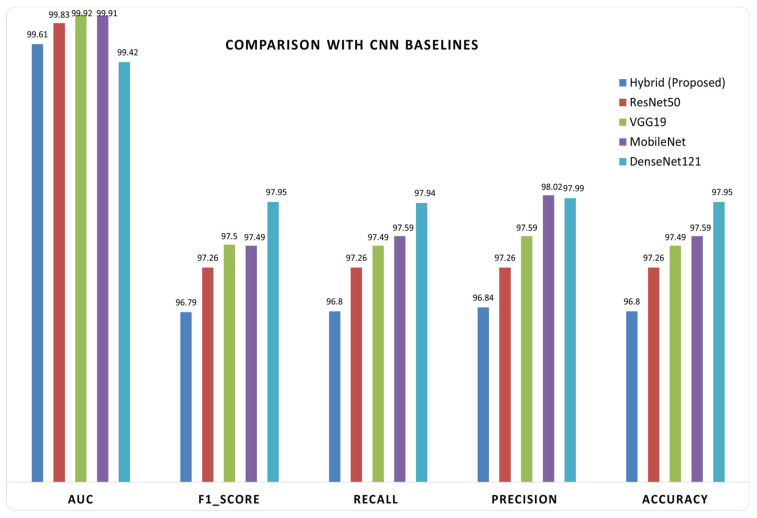
The proposed model with Keras CNN baselines.

Alongside benchmarking against the literature, a controlled comparison was conducted between our hybrid architecture, which utilizes an EfficientNetV2 backbone paired with a lightweight vision transformer (ViT) head, and established Keras CNN baselines, including DenseNet121, MobileNet, ResNet50, and VGG19, all employing a standard transfer learning head as shown in Figure 8. To ensure fairness, the data split and evaluation pipeline were standardized.

Using the BUSI dataset, we employed the precise fixed test split produced by the hybrid pipeline (20% originals per class, consistent class mapping) and stratified the remaining 80% into training and validation sets to maintain class balance. Each Keras baseline was trained on images resized to 224 × 224 using the model-specific preprocess_input, corresponding to the respective backbone, along with a lightweight GAP-plus-linear classifier. Training was conducted in two stages utilizing Adam and class-weighted categorical cross-entropy: (i) the backbone was frozen for 10 epochs at a learning rate of 1 × 10^−3^, followed by (ii) full fine-tuning for an additional 10 epochs at a learning rate of 1 × 10^−4^. All models underwent a single evaluation on the same held-out test set. Performance was quantified using standard multi-class metrics—accuracy, precision, recall, F1 score, and AUC, were reported to isolate the impact of the model architecture from variations in data partitioning or preprocessing. Table 6 illustrates a comparison between the proposed hybrid model and various baseline CNN architectures. The suggested EfficientNetV2–ViT model attained an accuracy of 97.95%, equaling ResNet50 and surpassing VGG19, MobileNet, and DenseNet121. The hybrid model exhibited competitive performance in weighted precision, recall, and F1-score, with only slight deviations when compared to ResNet50. While ResNet50 and VGG19 attained marginally superior AUC values of 99.91% and 99.92%, respectively, the suggested model sustained a robust AUC of 99.42%. The results demonstrate that the hybrid model achieves comparable efficiency across all evaluation metrics while providing improved feature representation by integrating local and global information, thus establishing a balanced and effective method for breast ultrasound categorization.

## 7. Discussion

The results demonstrate that the proposed hybrid EfficientNetV2–vision transformer (ViT) model outperforms both the EfficientNetV2-only and ViT-only baselines in the context of accuracy, stability, and efficiency. Specifically, the EfficientNetV2 backbone achieved 80% accuracy, the ViT-only model reached 89.5%, while the hybrid architecture attained 97.95%, demonstrating that the combination of convolutional and transformer components provides clear complementary advantages.

The EfficientNetV2 only model gets challenged when analyzing long-range dependencies, and contextual relationships, EfficientNetV2 only model captures fine texture spatial information however may misclassify when lesions are diffused or overlap. Conversely, the ViT-only model efficiently analyzes global reasoning, however has challenges with analyzing ultrasound images when the images are noisy and have low contrast and the model is limited on biases.

The devised hybrid model efficiently combines both EfficientNetV2 and ViT. EfficientNetV2 is able to identify local features and provide self global attention. EfficientNetV2 and ViT fusion will improve evaluation metrics on hybrid models and contextual information.

Additionally, the hybrid model needs less operational costs compared to accessing EfficientNet only model. This is observed with the token compression model which reduces the attention token count from 197 to 10. With this, the self attention complexity is low, and the GPU workload for training and inferring is low. With ultrasound images having simple global features, less operational costs will improve the model more.

The chosen configuration (pooling factor = 2 and shallow transformer depth) illustrates a compromise between retaining diagnostically relevant features and balancing the expenditure associated with computation. In this instance, the increase in tokens is anticipated to increase the related expenses, and is therefore not likely to improve performance. While a comprehensive ablation study may elucidate these design decisions further, the proposed configuration is indicative of a reasonable balance between performance and efficiency. Therefore, the model is suitable even for clinical settings with limited resources and real-time requirements as it maintains a reasonable balance between accuracy, robustness, interpretability, and efficiency.

The incorporation of Grad-CAM visualizations and the subsequent quantitative gains, allow for the locating of discriminative tumor boundary regions, thereby contributing to an improvement in qualitative interpretability. These visual explanations match with the clinical data, and consequently, they increase the certainty in the model’s reasoning and support the model integration in the computer-aided diagnosis process.

Moreover, the model’s learned feature representations were proven to be structured t-SNE visualizations further demonstrate the separability and ability. Additionally, its discriminative ability t-SNE visualizations further demonstrate the model’s learned feature representations to be structured and separable, reinforcing its robustness and discriminative ability.

There are several limitations concerning the promising findings of this study. First, the proposed model has only been tested with one publicly available dataset (BUSI), which may restrict the extent of its generalizability. Future efforts will focus on the validation of real clinical data as well as multicenter datasets in order to enhance robustness across varying scenarios.

Moreover, in spite of the fact that the proposed model exhibits promising performance in terms of inference, the entire development and evaluation pipeline is still quite time-consuming. This is attributed to multiple training cycles, the aforementioned hyperparameter adjustment, and cross-validation which cumulatively multiply the time and computational cost of the experiments. Consequently, the model is indeed capable of performing practical inference, yet more optimizations are warranted to alleviate the time burden associated with the entire training and validation pipeline.

## 8. Conclusions

We propose a hybrid deep learning architecture for classifying breast ultrasound images. It uses EfficientNetV2-RW-S as a feature extractor and a vision transformer (ViT) encoder. To lower the computational requirements of self-attention, this model substitutes raw ViT patch embeddings for pooling-based feature tokenization. This method of token-compression allows for attention-preserving spatial and contextual information, while significantly reducing the attention complexity. In addition, the transformer only operates on ten tokens, achieving a sufficient trade-off between classification performance, computation cost, and memory efficiency.

While using our model on the BUSI dataset, we obtained an accuracy of 97.95%, a precision of 97.99%, a recall and F1-score of 97.95%, and an AUC of 99.42%. Outperforming both EfficientNetV2-only and ViT-only baselines suggests that ViTs, in addition to convolutional layers, may improve lesion classification performance in ultrasound images under noisy conditions. The small fold standard deviation also suggests that the model is performing consistently.

Model explainability is supplemented by Grad-CAM, which highlights clinically relevant lesion areas and reinforces predictive accuracy. The EfficientNetV2–ViT model also has the best trade-off between computation, explanation, and accuracy. This model is optimal for use in breast cancer screening and decision aid technology.

Future work will focus on validating the proposed approach across multicenter and cross-dataset ultrasound cohorts to improve generalizability. In addition, deployment readiness will be enhanced through model optimization techniques such as pruning, quantization, and knowledge distillation. More advanced attention-based interpretability methods will also be investigated to further improve clinical transparency and trust.

## Figures and Tables

**Table 1:** Model training parameters and system specifications.

Item	Value
Dataset	BUSI Breast ultrasound (3 classes: benign, malignant, normal)
Data split	80% training and validation, 20% testing
Cross-validation	Stratified 5-fold
Epochs	50 with early stopping (patience = 6)
Batch size	32
Input image size	224 × 224
Patch size	32
Optimizer	AdamW
Learning rate	0.0001
Loss function	Focal Loss (*α* = 0.9, *γ* = 2)
CPU	Intel Core i7-13620H
GPU	NVIDIA GeForce RTX 4060 (8 GB, 75 W)
Operating system	Windows (64-bit)
Software environment	Python, PyTorch 2.5.1

**Table 2:** Classification performance metrics of the proposed model on the BUSI dataset.

Class	Precision (%)	Recall (%)	F1-Score (%)	Support
Benign	96	99	97	175
Malignant	99	97	98	210
Normal	99	96	98	54
Accuracy		97.95		438
Macro Avg	98	97	97	439
Weighted Avg	98	98	98	439

**Table 3:** Cross-validation performance (mean ± standard deviation) of the proposed model.

Metric	Mean ± std
Accuracy	96.86% ± 1.21%
Precision	96.96% ± 1.15%
Recall	96.86% ± 1.21%
F1-Score	96.87% ± 1.21%
MCC	91.94% ± 6.91%

**Table 4:** Confusion matrix of the proposed model.

True/Predicted	Benign	Malignant	Normal
Benign	0.99	0.01	0.01
Malignant	0.03	0.97	0.00
Normal	0.00	0.00	1.00

**Table 5:** Computation complexity of proposed model.

Metric	Value
Total parameter	28.26 M
Token count	10
Training peak memory	~3.2 GB
Inference peak memory	~1.7 GB
Inference latency	~0.054 s/batch
Inference throughput	~595 images/s

**Table 6:** Comparative results of the proposed hybrid EfficientNetV2 + ViT model versus established Keras CNN baselines.

Model	Accuracy	Precision	Recall	F1-Score	AUC
Hybrid_EffV2-ViT	97.95	97.99	97.94	97.95	99.42
ResNet50	97.95	98.02	97.95	97.94	99.91
VGG19	97.49	97.59	97.49	97.5	99.92
MobileNet	97.26	97.26	97.26	97.26	99.83
DenseNet121	96.8	96.84	96.8	96.79	99.61

## Data Availability

The original data presented in the study are openly available in Kaggle at https://www.kaggle.com/datasets/aryashah2k/breast-ultrasound-images-dataset (accessed on 1 January 2025).

## Abbreviations

The following abbreviations are used in this manuscript:

BUSI	Breast Ultrasound Images
MBconv	Mobile Inverted Bottleneck Convolution
Grad-CAM	Gradient-weighted Class Activation Mapping
